# Insect α-Amylases and Their Application in Pest Management

**DOI:** 10.3390/molecules28237888

**Published:** 2023-12-01

**Authors:** Beibei Wang, Daye Huang, Chunxia Cao, Yan Gong

**Affiliations:** National Biopesticide Engineering Research Centre, Hubei Biopesticide Engineering Research Centre, Hubei Academy of Agricultural Sciences, Wuhan 430064, China; wangbei_zju@163.com (B.W.);

**Keywords:** insect α-amylases, sequences, enzyme properties, α-amylase inhibitors, pesticides

## Abstract

Amylase is an indispensable hydrolase in insect growth and development. Its varied enzymatic parameters cause insects to have strong stress resistance. Amylase gene replication is a very common phenomenon in insects, and different copies of amylase genes enable changes in its location and function. In addition, the classification, structure, and interaction between insect amylase inhibitors and amylases have also invoked the attention of researchers. Some plant-derived amylase inhibitors have inhibitory activities against insect amylases and even mammalian amylases. In recent years, an increasing number of studies have clarified the effects of pesticides on the amylase activity of target and non-target pests, which provides a theoretical basis for exploring safe and efficient pesticides, while the exact lethal mechanisms and safety in field applications remain unclear. Here, we summarize the most recent advances in insect amylase studies, including its sequence and characteristics and the regulation of amylase inhibitors (α-AIs). Importantly, the application of amylases as the nanocide trigger, RNAi, or other kinds of pesticide targets will be discussed. A comprehensive foundation will be provided for applying insect amylases to the development of new-generation insect management tools and improving the specificity, stability, and safety of pesticides.

## 1. Introduction

Amylase is one of the most common hydrolases in nature that can hydrolyze starch, glycogen molecules, and related glucans into a variety of products, including dextrins and smaller polymers composed of glucose units [1]. It is widely distributed and coexists in all species, such as humans [2], pigs [3], chickens [4], oysters [5], plants [6], fungi [7], bacteria [8], and insects [9,10,11]. Based on the different types of hydrolysate isomerization, amylase can be categorized as α-, β-, or γ-amylase, and each of them acts on different parts of the carbohydrate molecule. So far, only α-amylase (α-1,4-glucan-4-glucanohydrolases, EC 3.2.1.1) has been reported in insects [12].

The wide distribution, variety, and large quantity of insects indicate that they have amazing survival ability. A core challenge for insects’ survival is the huge changes in food availability, and, to cope with this challenge, insects have evolved various feeding preferences to obtain energy. These feeding preferences have also been conditioned based on the nutrient content provided by the food. For example, plant starch is a major source of carbohydrates for herbivorous insects. α-1,4glycosidic bonds in starch are hydrolyzed by α-amylase and converted into simple sugar units, providing energy for insects to survive and develop [13].

Previous studies have elucidated that α-amylases exist in different insect orders (Diptera, Coleoptera, Heteroptera, and Hymenoptera); these enzymes participate in the process of insects’ digestive adaptation to host plants to obtain the maximum amount of nutrients required for development and reproduction [14,15,16]. These studies on insect amylases are mainly focused on their sequences, structure, enzyme kinetics, evolution, interaction with amylase inhibitors, and transgenics with α-AIs. Nevertheless, the information and conclusions obtained from the basic research on insect amylases do not provide enough inspiration to facilitate applied research, and most of the available applied studies fail to figure out the safety and stability of pesticides and transgene plants. In recent years, scientists have been focusing more on these challenges and an accumulating body of research has focused on the application of insect amylases.

In this review, the sequences, structure, properties, and inhibitors of α-amylase in different insect orders are discussed and compared, accompanied by our comprehensive survey of insect amylase as a target of insecticide. A comprehensive foundation will be provided for the application of insect amylase as a desirable pesticide and new pest management strategies.

## 2. Sequences and Structure of Insect α-Amylases

In recent years, significant development has taken place in the study of the sequences and architectures of insect α-amylases due to the advances in experimental technology including multi-omics sequencing, molecular biotechnology, and silico studies. For example, the number of known amylase genes has exploded, and innumerable insect amylase sequences have been published in various genome databases.

Available data indicate that all insect amylases are nearly the same size, i.e., coding sequences contain about 1500 nucleotides [17] and the amylase sequences in different species are highly conserved in some regions. During long-term evolution, the amylase sequences also mutate between related species, which makes amylases differ significantly from inexact substrate preferences. Jing et al. [18] have demonstrated that α-amylase showed 94% similarity between the *Conogethes pinicolalis* and its sibling species *C. punctiferalis* and that α-amylase gene mutations occur in non-homologous conserved regions. Mutations of this type do not cause structural changes, but they may affect changes in α-amylase expression levels and enzyme activity.

**Figure 1:**
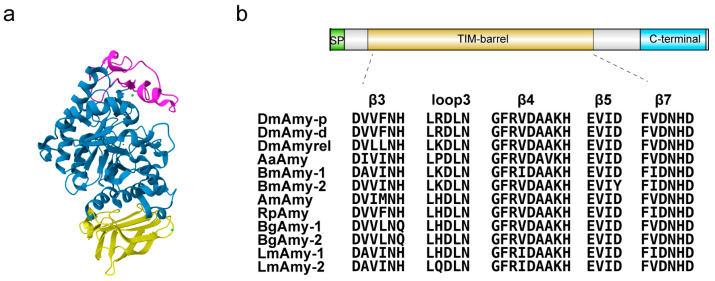
(**a**) Three-dimensional structure of a *Drosophila melanogaster* α-amylase (Amy-P) (PDB code 8or6): domain A in blue, domain B in pink, domain C in yellow; green spheres present strontium ion; the red sphere presents a chloride ion. (**b**) Schematic diagram of the domain composition of Amy-P and alignment of α-amylase protein sequences of model insects. Signal peptide (SP), catalytic domain ((β/α)8-barrel domain), and C-terminal all-beta (C-terminal) domains are boxed with the green, brown, and green backgrounds, respectively. Conserved amino acid sequences in 12 insect α-amylase proteins, including 3 from *Drosophila melanogaster*—DmAmy-p(AAF57896.1), DmAmy-d (AAF57894.1), and DmAmyrel (AAF57971.1); 1 from *Aedes aegypti*—AaAmy (AAB60934.1); 2 from *Bombyx mori*—BmAmy-1 (XP_021208434.1) and BmAmy-2 (XP_004924134.1); 1 from *Apis mellifer*—AmAmy (NP_001011598.1); 1 from *Rhodnius prolixus*—RhAmy (JAA77077.1); 2 from *Blattella germanica*—BgAmy-1 (ABC68516.1) and BgAmy-2 (AGV15452.1); and 2 from *Locusta migratoria*—LmAmy-1 (Lmig011911.1) and LmAmy-2 (Lmig011912.1).

Although some amino acid sequences of insect α-amylases are open in many databases, only three insect α-amylases with a known three-dimensional structure (3D structure) have been reported, i.e., *Tenebrio molitor* larva α-amylase (TMA) [19,20], *Ephestia kuehniella* (Lepidoptera) isoenzyme 3 (EkAmy3) [21], and *Drosophila melanogaster* α-amylase (DMA) [22]. According to a sequence-based classification, α-amylases belong to the largest family of glycoside hydrolases GH13 [23]. In general, the α-amylases and other GH13 family members are three-domain proteins in a 3D structure: domain A, consisting of the main catalytic (β/α)_8_-barrel; domain B, a variable-length loop located between sheet β3 and the α3 helix of domain A; and domain C, a Greek key motif in the C-terminal position [13,24]. Figure 1a shows the three-dimensional structure of *Drosophila melanogaster* α-amylase (PDB 8OR6, [22]) with three domains. The α-amylases utilize a reaction mechanism that retains configuration, have 4–7 conserved sequence regions (CSRs) and catalytic machinery in common, and adopt the catalytic domain of (β/α)8-barrel [13]. Figure 1b shows the conservatism of conserved sequence regions and the catalytic residues of several model insect α-amylase sequences. A triad of acidic groups constituting catalytic residues is strictly conserved in the active sites of the α-amylase enzyme family: an Asp as a catalytic nucleophile, a Glu as a proton donor, and an additional Asp involved in substrate binding [24].

The functional characteristics of enzymes are closely related to protein motions and conformational changes. Cipolla et al. [25] have reported a striking continuity in the functional properties of α-amylase regarding its structural stability and the thermal state of the source organism. Hámori et al. [26] used SUMA program calculations for the first subsite map of an insect α-amylase and first reported the characterization of the active center of Colorado potato beetle (*Leptinotarsa decemlineata*) α-amylase (LdAmy), which elucidates the action pattern and product specificity of LdAmy. They also revealed that the binding region in LdAmy has six subsites, which is similar to human salivary α-amylase and porcine pancreatic α-amylase.

Insect α-amylase sequence research will be of great significance for understanding the internal mechanism of amylase and the role it plays in related signaling pathways. It contributes to the development of new pesticides. It also gives novel insights into species evolution and adaptation to various diets. Unfortunately, there are currently few studies on the structure of insect amylases.

It is interesting to note that α-amylase paralog Amyrel presence in true flies (Diptera Muscomorpha) has been classified as a CAZy family GH13 glycoside hydrolase based on its primary structure, and Amyrel is unique among animals as it possesses both hydrolytic α-amylase activity (EC 3.2.1.1) and 4-α-glucanotransferase (EC 2.4.1.25) transglycosylation activity [27].

## 3. Multigene Family of α-Amylase in Insects

A gene family is defined as a group of genes encoding different proteins, which are considered to have evolved from a single ancestral gene due to their high degree of sequence similarity [28]. α-Amylases usually form multigene families in animals, plants, fungi, and bacteria [22]. Amylase gene copies were reported in many insect species and the copy number varied from one to more than twelve [17]. For example, fourteen α-amylases have been identified in *Lutzomyia longipalpis* [23]. The phenomenon of multiple copies in many species of insects may be because most insects rely on polysaccharides for energy, and the breakdown of polysaccharides depends on the activity of amylase. Therefore, multiple copies of the amylase gene in insects give the insect an evolutionary advantage. The comparison of amylase sequences in *Drosophila melanogaster* indicates that repeated gene transformations lead to the co-evolution of gene pairs [29]. So, α-amylase can serve as a model for the evolution of multiple gene families [30].

Dietary flexibility may increase with the growing number of gene copies. Mythimna genus pests were observed as a tandem repeat expansion of α-amylase genes, which may promote the digestion of carbohydrates and exacerbate the damage to crops. Furthermore, tandem repeat expansion of α-amylase genes gives insects strong digestive ability [31]. Amylase activity varies in different insects. In the tobacco hornworm, *Manduca sexta*, the mRNA level of α-amylase-1 is about ten times higher than that of α-amylase-2 during the feeding stage [32]. However, the links between α-amylase gene copies and dietary habits are still unknown.

The widespread presence of multigene families of amylases often results in different kinetic parameters and regulatory characteristics among different amylases, and these differences allow the diversity of the metabolism of organisms to meet the special needs of specific tissues or different developmental stages. For example, seven α-amylase species have been found in the midgut of larvae of *Cerambyx cerdo*. The generation of these various subtypes of amylase might be caused by wide geographical distribution and feeding habits [33]. *Eurygaster integriceps* salivary gland complexes have at least five digestive enzymes isoamylases [34].

The duplication of amylase genes helps to evolve new functions and may also result in the production of isozymes of amylases. α-Amylase isoforms have also been reported in some insects [35], but there is a lack of research on the function and expression specificity of these enzymes. Most amylases are present in the gut of insects, and multigene replication can also lead to diversity in the expression location and function of amylases. Some changes in tissue-specific amylases and their consequent functions have been reported [17].

Several α-amylases have been detected in the non-digestive tract, which proves that α-amylase is not expressed only in insect midguts and salivary glands (Table 1). Several studies described the function of α-amylase that was tested in other tissues. For instance, α-Amylase found in forager bee (*Apis mellifera* L.) hypopharyngeal glands can convert nectar into honey [36], and it is also a candidate protein encoded by methylated genes, which have been used to demonstrate that DNA methylation may play important roles in the activation of the hypopharyngeal glands of overwintering honeybees [37]. Moreover, the α-amylase expressed in the male German cockroach, *Blattella germanica* L., tergal glands has been proven to function as a nuptial feeding stimulant [11,38]. Gene copies are common in parasitic wasps, such as *Ampulex compressa* and *Nasonia Vitripennis* [39]. Some multi-copy amylase genes showed tissue-specific and functional differentiation. In parasitic wasps *Pteromalus puparum*, three α-amylase (*PpAmys*) have been identified: *PpAmy1* is intestinal amylase, *PpAmy2* acts in muscle metabolism, and *PpAmy3*, specifically expressed in the venom gland of the parasitoid, could affect the host metabolism that supports the development of parasitic wasp offspring. Furthermore, phylogenetic results show gene duplication in *PpAmy2* and *PpAmy3* [40,41]. The oral secretions of larvae of lepidopteran stem borers’ α-amylase can mediate host acceptance of oviposition in *Cotesia flavipes*, and these secretions allow the female parasitoids to distinguish between host and non-host larvae [42].

## 4. Properties of Insect α-Amylases

The active site of the enzyme is usually composed of ionizable groups, and only when these groups keep the appropriate ionic form can they maintain the conformation of the active site, for which an appropriate pH value is one of the key factors. The optimum pH is varying in different species of insects to maintain α-amylase activity.

The optimal pH for α-amylase activity in Lepidoptera is extremely alkaline, while the optimal pH for α-amylase activity in most Coleoptera and Hemiptera insects is acidic to neutral, that is, 4.0–7.0 [35]. For example, three digestive α-amylase activities of flour moth *Ephestia kuehniella* were observed in the alkaline range, and the maximum pH observed was between pH 9 and pH 10 [21]; the optimal pH value of α-amylase in *Helicoverpa armigera* (Hübner) is 10.0 [43]. In addition, the optimal pH for the α-amylase activity of Coleoptera, Diptera, and Hymenoptera insects is a weakly acidic pH, which makes them sensitive to α-amylase inhibitors in legumes [44]. The optimal condition for *Sitophilus oryzae* α-amylase activity is a pH of 5.0 [45]. Moreover, the optimal pH for α-amylase in some Hemiptera insects is alkaline; for example, the optimal pH for α-amylase in *Andrallus spinidens* is 9.0 [46].

The optimum temperature for maintaining α-amylase activity in insects is between 30 °C and 60 °C, and α-amylase activity drops sharply when the temperature reaches above 60 °C. A study showed that α-amylase from the midgut of *Alphitobius diaperinus* (Coleoptera: Tenebrionidae) larvae reached an optimal temperature of 45 °C. It maintained 34.6% activity after being kept at 60 °C for 5 min, and activity dramatically decreased to 23% when placed at 80 °C for 1 h. The presence of high levels of Ca^2+^ (2 mM) and Na^+^ (100 mM) ions was also shown to decrease enzyme activity [47]. The *H. armigera* α-amylase optimum temperature is 50 °C [43].

In addition, the activity of α-amylase is also different when the substrates are various. It has a substrate preference and can accurately and efficiently recognize specific types of catalytic substrates, which play a beneficial role in helping metabolic systems function well. For example, in *Morimus funereus*, maximum amylase activity has been achieved when the catalytic substrate is horseradish starch, and activity is undetectable with potato starch [48]. The *H. armigera* α-amylase exhibited high activity when the substrates were starch and amylopectin; if they were dextrins, the activity was poor [43].

## 5. The Regulation of Insect α-Amylases Expression and Activity

Given the vital role that α-amylase plays in insect carbohydrate metabolism and physiological and biochemical processes, the molecular model of starch digestion, the absorption of glucose molecules, and the regulation of insect α-amylase expression and activity have been extensively studied.

In *Musca domestica*, starch is firstly digested by amylase and maltase in the anterior midgut and then the resulting glucose units are absorbed in the midgut. Sugars and glycogen are released by bacterial and fungal cells in the middle of the midgut and the remaining starch is digested by amylase and maltase from the end of the midgut to the middle of the hindgut, and the resulting sugar is absorbed by the hindgut [49].

Many genes and signaling pathways have been detected in the modulation of amylase activity. In 1978, Abraham and Doane reported a tissue-specific gene *map* in *Drosophila melanogaster* that can control amylase activity in the posterior midgut of adult females but has no effect on amylase activity in the anterior midgut [50]. Another study on fruit flies described that the presence of polymorphic regulatory genes affects the expression levels of amylase proteins [51]. In the cockroach (*Periplaneta americana*) midgut, amylase activity can be stimulated by adipokinetic hormones (AKHs) [12]. As for *Ostrinia furnacalis*, larval feeding is regulated by neuropeptide F in the midgut via the insulin signaling pathway. Furthermore, the genes *pi3k* and *mtor* positively regulate the activity of α-amylase by recruiting the transcription factor c-Myc, which can bind to the promotors of this enzyme [52].

Changes in α-amylase activity may be influenced by the habitat, feeding, and developmental stages of insects. Therefore, α-amylase is considered an ideal gene product for studying gene expression patterns during insect development. For example, amylase activities vary when *Hyphantria cunea* larvae are fed with leaves of different preference host plants, which leads to changes in the body weight, food intake, food conversion rate, and food utilization rate of *H. cunea* larvae [53]. Similar studies have shown that variations in the nutrient properties of food types cause changes in α-amylase activities. For instance, western flower thrips, *Frankkliniella occidentalis*, fed on different foods experienced changes in α-amylase activity in various generations, i.e., after feeding on rose petals, α-amylase activity decreased significantly in the F1 generation and increased significantly in the F2 generation [54]. These results suggest that insects improve the efficiency of food utilization and absorption of host plants by regulating the activity of digestive enzymes in larvae.

Enzyme activity can also change when artificial feed is used as a food source. Studies have shown that α-amylase activity significantly increased in an artificial diet group during the 2nd- and 3rd-instar in silkworm (*Bombyx mori*) compared to a mulberry leaves group; the reason may be that the artificial feed contained more maize starch [55]. In the case of *B. mandarina*, artificial selection forced the early ancestors of domesticated silkworms to raise the expression of amylase to digest starch more efficiently, resulting in larger bodies and a higher silk-producing capacity than their ancestors. Specifically, overexpressed α-amylase in *Bombyx mori* midguts (*BmAmy1*) could enhance the growth of silkworms, increasing the whole cocoon and cocoon shell weights [56,57]. Another study has suggested that artificial diet carbohydrate and protein contents induce changes in nutritional efficiency, development, and α-amylase activity in *Plodia interpunctella* (Lepidoptera: Pyralidae) [58]. The characterization and activity of insect α-amylase when reared on different poultry diets are various. These studies will help researchers gather information and find new tools to control insects.

Digestive physiology also mediates the adaptation of insects to multiple environments. The activity of α-amylase has significant differences in diapausing and non-diapausing *Eurytoma plotnikovi* (Hymenoptera: Eurytomidae) larvae [59]. Similarly, Dmochowska et al. have documented that at the end of the diapause of the red mason bee, *Osmia rufa* L. (Hymenoptera: Megachilidae), amylase activities are significantly reduced [60]. In wheat pest *Eurygaster integriceps*, diapause development is closely related to enzyme activity [61].

## 6. Insect α-Amylase Inhibitors

Flora has undergone a process of evolution, developing the capability to synthesize a diverse array of natural metabolites as a means of safeguarding itself from potential threats, including insects, predators, microorganisms, and environmental factors such as temperature, pH, humidity, salinity, and drought. α-Amylase inhibitors, classified as plant-derived toxic proteins, are produced by various parts of plants, including roots, tubers, stems, fruits, buds, and leaves [62]. For example, the banana pseudo-stem weevil, *Odoiporus longicollis* Olivier, larva gains the ability to resist the toxicity of stigmasterol-3-O-glucoside (SOG), sulfoquinovosyl diacylglycerol (SQDG), and betulinic acid (BA) produced by pseudo-stems through synthesizing excess α-amylase [63]. In addition, an α-amylase inhibitor extracted from *Moringa oleifera* leaf has insecticidal effects on *Callosobruchus maculates* insect larvae [64]

α-Amylase inhibitors (α-AIs) have been classified by Richardson into six classes according to their tertiary structure: lectin-like, knottin-like, cereal-like, Kunitz-like, γ-purothionin-like, and thaumatin-like [16]. These classes of inhibitors show significant structural diversity, resulting in different inhibition patterns and specificities against different α-amylases. For example, the common bean α-AI1 in complex with TMA [19] was used to elucidate the inhibitory mechanism of lectin-like inhibitors; structural analysis shows that the two hairpin rings of α-AI1 are inserted into the TMA reaction site, blocking substrate binding and establishing a hydrogen bonding network with residues in the substrate binding region [16]. As for knottin-like α-amylase inhibitors, they achieve inhibition by blocking catalytic sites, and the inhibitors bind in gaps between active sites and interact with catalytic residues in the A and B domains of α-amylase [65]. The specificity of inhibition is an important issue because the introduced inhibitors cannot adversely affect the plant’s α-amylase and cannot affect the nutritional value of the crop [16].

Plant seeds are an important source of α-amylase inhibitors [66]. α-Amylase inhibitors in cereal grains and legume plants can regulate the endogenous α-amylase activity and the immune response of pathogens and parasites [66]. Lectin-like inhibitors include two types: α-AI1 and α-AI2; they are derived from common white, red, and black kidney beans. White bean protein and rapeseed protein extracts can induce larval lethality by modulating the expression of the α-amylase gene in Colorado potato beetle [67]. Genetically modified (GM) seeds of chickpeas contain a bean α-amylase inhibitor (αAI-1) that is immune to a variety of bruchid pests and harmless to some important groups of non-target insects, including Hymenopteran bruchid parasitoids. GM seeds and natural enemies can be combined for pest management [68]. Biological and environmental safety are two important indicators when considering whether GM seeds can be applied in farmland. An α-amylase inhibitor (α-AIC3) expressed by using the *Nicotiana benthamiana* expression system could inhibit up to 100% of cotton boll weevil, *Anthonomus grandis*, amylase biological activity, whereas it exhibits no effect on the α-amylase of two non-pathogenic insects: *Apis mellifera* amylase and *Spodoptera frugiperda* amylase [69].

The major α-amylase inhibitor (AAI) in the seeds of *Amaranthus hypocondriacus* is specific against insect amylases and cannot affect human or mammalian enzymes. It is classified as a knottin-like protein according to its structure [70]. Rane et al. have identified three previously unidentified knottin-like α-AIs: *Amaranthus hypochondriacus* (AhAI2), *Alternanthera sessilis* (AsAI), and *Chenopodium quinoa* (CqAI), which have been proven to have the specific inhibitory activity of Coleopteran α-amylases and no cross-reactivity with mammalian α-amylases [71].

Cereal-like inhibitors (CTIs) have various degrees of effects on the digestion of mammalian and insect digestive α-amylases. Their inhibitory function of α-amylases activity is influenced by the substrate [72]. For instance, an amylase inhibitor isolated from whole flour extracts of tetraploid wheat has an inhibitory protein for human salivary amylase and *Tribolium castaneum*, *Tenebrio molitor*, *Sitophilus oryzae*, and *Ephestia kuehniella* α-amylase. The inhibition of the α-amylase of Coleoptera was stronger than that of human salivary α-amylase [73]. Cereal proteins are the main nutritional proteins of *Tenebrio molitor* L. larvae; the impact of CTI in food is proven to be correlated to the expression of digestive enzymes, whereas it is currently difficult to determine their potential impact on the developmental characteristics of larvae [74].

The well-studied α-amylase inhibitor in the kunitz class is the barley α-amylase/subtilisin inhibitor (BASI). The biochemical characteristics of various barley cultivars seeds and the expression levels of gut digestive enzymes have an impact on the fitness of *Rhyzopertha dominica* [75].

The well-learned inhibitor of the thaumatin type is from maize, a bifunctional inhibitor from *Zea mays*. [76,77]. γ-Purothionin-type inhibitors include three isoforms: SIα1, SIα2, and SIα3, which are extracted from *Sorghum bicolor*. These inhibitors have strong inhibitory effects on α-amylase in the gut of locusts and cockroaches, less inhibitory influence on α-amylase in *Aspergillus oryzae* and human saliva, and no significant inhibitory effect on α-amylase in the pancreas of pigs [78].

Wheat kernels are richly endowed with α-amylase inhibitors that affect both insects and mammals. Similar to the porcine pancreatic α-amylase (PPA) active site, according to the pattern of early hydrolysis products of 4,6-O-benzylidene-modified substrate (BzG7PNP), *Leptinotarsa decemlineata* α-amylase (LDAmy) may have three glycon- and two aglycon-binding sites. The similarity between LDAmy and PPA enhances the utilization of known mammal α-amylase and the possibility of amylase inhibitors to protect potato plants from the attack of Colorado potato beetles [79]. In some cases, α-amylase inhibitors only work on mammalian α-amylases, and sometimes only on insect α-amylases. Pest-specific α-amylase inhibitors provide potential tools for plant defense. In summary, a better understanding of the structural basis of the suppression spectrum can enable the rational design of mutants with more ideal characteristics.

New promising synthetic inhibitors are yet to be developed [80]. Phyto defensins are an important superfamily of innate immune proteins that have strong inhibitory effects on human infectious diseases. It has antifungal and antibacterial activities and inhibitory activities against insect amylase [81]. Plant defensins are a large family of 45–54 amino acid residues and cationic proteins present in different parts of the plant, such as roots, seeds, leaves, flowers, and stems [82,83]. The most common activity is their antifungal effect; anti-bacterial and anti-virus function; protein, α-amylase, and protease inhibitory action; and ion channel blocking effect.

Plant defensins remain structurally stable at extreme temperatures and pH and are generally non-toxic to mammalian cells, making them the most promising candidate for pest management. Therefore, revealing the relationship between the structure and function of plant defensins is helpful for their application [84]. For instance, Defensin *PsDef1* from gymnosperms *Pinus sylvestris* (Scots pine) inhibits the activity of α-amylase from pine beauty moth, *Panolis flammea* [85], and *PsDef2* shows a stronger inhibitory effect on α-amylases from May’s beetle larva, cabbage butterfly, and darkling beetle than that of *PsDef1* [84]. The widespread and diverse presence of defensins in the plant kingdom suggests that these kinds of proteins may be a potential source of antimicrobial activity, with broad prospects for agricultural biotechnology and pharmaceutical applications [81].

Some non-proteins also have inhibitory activities on amylase, such as acarbose, isoacarbose, hibiscus acid, acarviosine-glucose, and cyclodextrins [16]. Some bioactive compound–drug interactions could improve the therapeutic properties of acarbose, eg., gallic acid and its polymeric form; tannic acid could increase enzymes’ inhibitory effects and the antioxidant properties of acarbose in vitro [86].

The characteristics of nonproteinaceous inhibitors have attracted people’s interest in the medical field; however, it is much more difficult to produce insect-resistant transgenic plants because their production is very complex and involves multiple metabolic pathways. In the field of insect-resistant genetically modified organisms, protein inhibitors encoded by a single gene have more advantages. Transgenic plants that produce α-amylase inhibitors are effective alternatives to chemical pesticides, which may lead to the development of crop varieties that are resistant to major target pests. Amylase inhibitors are reported to be a source of insecticidal transgenics and have been used to develop insect-resistant crops [87]. Different grain varieties and genetically modified grains may contain compounds such as amidohydrolase or protein inhibitors of proteolytic enzymes, which may enhance their resistance to pests by impeding their development. To avoid resistance and the adverse effects of chemical pesticides, alternative methods are urgently needed.

Despite the abundance and diversity of insect α-amylase inhibitors, how to utilize them is an important issue: α-amylase inhibitor recombinant proteins and transgenic plants are two efficient ways. In view of the need for convenient production with retention of the inhibitory activity of α-amylase, Giri et al. [88] combined a premature α-amylase inhibitor (PMAI) protein isolated from *Amaranthus hypochondriacus* with a complete signal peptide to express the recombinant PMAI in a bacterial expression system. The purified recombinant comprised strong amylase inhibitory (AI) activity against storage insect pest amylases such as *Tribolium castaneum* and no inhibitory effect on human salivary α-amylases. Sa et al. [89] transformed α-amylase analogous mutant inhibitors (αAIs) obtained from common bean (*Phaseopusp vulgaris*) into cotton (*Gossypium hirsutum*). The transgenic plant was proven to have insecticidal activity against the Coleopteran insect pest boll weevil *Anthonomus grandis*.

α-Amylase inhibitors exhibit differences in biochemical characteristics, enzyme catalysis, and regulatory mechanisms. In-depth structure analysis is helpful in understanding the interaction between amylase and α-AIs; thus, designing and exploring novel possible applications and synthetic routes are necessary. A deep understanding of the structural basis of the interaction between amylase and α-AIs will facilitate the design of structurally based inhibitors or the site-specific/saturation mutagenesis of existing inhibitors to regulate their activity and selectivity. In addition, the information can also be used for peptide simulation or the design of amylase inhibitors based on small molecule drug groups [90]. Moreover, α-AIs polypeptides can also be designed to inhibit the activity of α-amylase in specific insects. These studies lay the foundation for the design of inhibitors that are specific and safe for the ecosystem.

## 7. Insect α-Amylase as a Pesticide Target

The digestion of food is essential for the survival and prosperity of insects. Therefore, α-amylase, one important type of insect digestive enzyme, has served as an appealing protein target to fight insect pests.

Nano-pesticide formulations are currently one of the hotspots in pesticide research. Nanopesticides have many advantages in prevention and control efficacy, absorption conductivity, and permeability compared to conventional pesticides [91]. Nanoparticles are usually classified into two categories: carrier nanoparticles and non-carrier nanoparticles. Compared with non-carrier nanomaterialization, the properties of carrier nanomaterialization depend on the carrier, which is more suitable for the development of modern pesticides [92]. The controlled release mechanisms that are currently available in nanopesticides include pH, temperature, light, and enzymes. In addition, the amylases, proteases, and cellulases in the gut of insects usually serve as triggers for these mechanisms, and α-amylase is one of the important triggers used in nanopesticides. Amylase in a pest’s digestive tract can degrade polysaccharides coated with nano-pesticides so that it has a slow and controlled release capability that could intelligently release according to the digestive environment of the pest. For example, Zhang et al. [93] constructed a pH- and amylase-response structure using ZIF-8 as solid supports and αcyclodextrin (α-CD) as a gatekeeper to the site-specific delivery of thiacloprid; it released quickly in pea aphid intestines because of the disintegration of ZIF-8 at low pH values and the degradation of α-CD by α-amylase. Thus, it could control pea aphids and was safe for earthworms. Similarly, Yang et al. [94] reported an insecticide delivery system based on β-cyclodextrin (β-CD)-anchored hollow mesoporous silica (HMS) nanoparticles. It exhibited dual response properties to pH and α-amylase excellently, and these properties could be applied to control *Spodoptera frugiperda* and reduce harm to non-target organisms such as zebrafish. In addition, different types of pesticides can be used for the preparation of nano pesticides. For instance, a novel redox and α-amylase dual stimuli-responsive pesticide delivery system was established by combining functionalized starch with biodegradable disulfide-bond-bridged mesoporous silica nanoparticles loaded with avermectin, which displayed a more durable control effect on *Plutella xylostella* larvae compared to avermectin emulsifiable concentrate [95]. Moreover, the biological synthesis of *Bacillus thuringiensis*-coated zinc oxide nanoparticles (Bt-ZnO NPs) has demonstrated significant effects on the hatchability, fecundity, and larval and pupal development period of *Callosobruchus maculatus* [96]. α-amylase can also work as a target gene; it could be knocked down with nanoparticle-encapsulated dsRNA conjugates to manage pests efficiently [97].

Furthermore, the accumulation of nanomaterials in edible plants, animals, and the environment has also caused concerns about human toxicology and ecotoxicity. Copper and zinc oxide nanoparticles (CuO and ZnO NPs, respectively), as the commonly used engineered nanomaterial productions, could impair α-amylase activity, thus inhibiting the food digestion and nutrient uptake of insects and resulting in poor fitness or eventual death. More seriously, it has the potential to inevitably influence all living things including non-target organisms such as silkworms due to possible mishandling and intensive nanomaterials [98].

Bio-pesticides are the best alternatives to synthetic pesticides. α-amylases also are targets of bio-pesticides. For example, Azukisapogenol triterpenoid saponins from *Oxytropis hirta* could be used as a kind of biopesticide to control pea aphids (*Acyrthosiphon pisum*); their suppression of α-amylase could be one sign of organelle damage in the midgut [99]. As a class of bio-pesticides, some plant-derived extracts have proven to inhibit amylases and are promising natural alternatives to control pests. For example, plant-derived extracts from *Magnolia grandiflora* (Magnoliaceae), *Schinus terebinthifolius* (Anacardiaceae), and *Salix babylonica* (Salicaceae) have the amylase inhibitory effect of *Spodoptera littoralis* (Boisd.) [100]. The inhibition of plant extracts on the amylase activity of pests was achieved through feeding by fumigation, contact, and ingestion [101,102,103,104,105,106,107]. The inhibitory activity of these compounds on α-amylase is partially due to their cyclic structure, which is similar to the substrate of α-amylase, thus binding to the catalytic site of α-amylase and inhibiting the enzyme.

Interestingly, amylase activity in insects remains unchanged or is upregulated in some pesticide treatments, but can still cause damage or death. Pyriproxyfen is a hormonal pesticide that can poison a non-target insect, silkworm, leading to prolonged silkworm larval instar and blocking the life cycle. After being treated with Pyriproxyfen, the activity of α-amylase was significantly increased, but the microvilli and goblet cells were severely ruptured in the midgut tissue of silkworms [108]. In addition, Phoxim can increase the activities of silkworm α-amylase, which disturbs the metabolism of carbohydrates [109]. Li et al. [110] reported adding a plant-derived extract—Toosendanin (TSN), from the root bark of *Melia toosendan*, after added it to the *Mythimna separata* Walker. The activities of larval midgut amylase did not change significantly, but the microvilli arrangement of midgut cells was disordered and the mitochondria in it increased irregularly.

RNA interference (RNAi) has become a widely used tool for pest control. α-amylase is the most promising target gene for the RNAi-mediated control of some pests [111]. Cotton boll weevil *Anthonomus grandis* feeding on α-amylase dsRNA has shown a larvae mortality rate of 60% and mortality in adults of 30% [112]. Both diet and injected delivered dsRNA could effectively silence the α-amylase gene, but dsRNA injection bioassay was more effective than ingestion in *Helicoverpa armigera* [113]. These pesticides also have certain effects on the amylase activity of non-target pests. Sublethal spinetoram and glyphosate exposure can significantly decrease gut α-amylase activity in *Bombus terrestris*, which impairs their health [114]. In recent years, more and more different kinds of agents have used amylases as the target of pest control [115,116,117,118], which leads to the metabolic disorder of pests by reducing or increasing enzyme activity. Some of the literature is listed in Table 2. It should be noted that the lack of specificity of pesticides that are constructed on targeting amylases is an urgent problem yet to be solved.

## 8. Insect α-Amylases and Human Health

The cost and time effectiveness, high productivity, ease of modification, and optimization of the microorganism α-amylase make microbial-sourced α-amylase an ideal product for various large-scale utilizations, which has gained much attention in the industry. In recent years, with the growing developments in biotechnology and the increasing interest from both the scientific and industrial communities, the applications of α-amylase have been widened to other fields such as the clinical and medical fields.

α-amylases have been studied in the field of human health. It has been reported that α-amylase-inhibitors may be beneficial in treating type 2 diabetes [119,120]. An in vitro study has shown that different plants, mainly those traditionally used to treat diabetes in Africa or Europe, can inhibit α-amylase, i.e., a 90.0% inhibition of α-amylase activity was detected using the extract of *Tamarindus indica* leaves [121]. In addition, bee pollen has strong antioxidant activity and effective inhibition of α-amylase and α-glycosidase; these properties may support bees as a potential product for use in food formulations in the nutritional health sector, for example, functional and bioactive ingredients [122].

α-amylase is also a known allergen in several insects [123]. Insect exposure leads to high levels of sensitization among employees, and α-amylase is the culprit in some occupational mealworm allergy cases [124,125]. α-amylases exhibit a high degree of sequence similarity among mites, insects, and mammals, raising the possibility of being potential cross-reactive IGE-binding allergens [126]. Ric c1, an allergenic protein from castor oil plants (*Ricinus communis*), is an insect α-amylase inhibitor that has become an occupational allergen, and Pacheco et al. have used the point mutations to support our continuing efforts to produce transgenic hypoallergenic castor oil plants and develop an immunotherapeutic agent for allergy prevention [127].

Although amylase is widely distributed in plant, animal, and microbial species, only amylases in fungi and bacilli have made a significant contribution to the industrial market. It is a laborious, inefficient, and multi-step procedure to isolate and purify enzymes from small insects, and there is very little information about raw-starch enzymes for most of the insect species. However, allogenic overexpression offers the possibility to transcend these problems [45]. For example, a recombinant α-amylase from rice weevil can be efficiently expressed in *Saccharomyces cerevisiae*, and an insect amylase-based preparation, in a mixture with commercial glucoamylase, was used as an amylolytic agent in the processes of raw starch-to-ethanol production by wild-type ethanologenic yeasts [128].

Insect feed has a high protein conversion rate, beneficial nutritional components, and low greenhouse gas emissions. This might provide a solution for the growing global demand for protein in animal feed and human consumption. The selection and optimization of artificial feed formula to improve the activity of amylase has a positive effect on the physiological indexes of insects.

## 9. Conclusions and Perspective

The sequences and characterization of insect α-amylases and the effect of α-amylase inhibitors will contribute to providing more fundamental information in the search for pest control tools. Nonetheless, there are four main problems in insect α-amylases research: (1) basic research of insect α-amylases does not combine well with application research; (2) the conclusions of interaction studies between insect α-amylases and amylase inhibitors is ambiguous; (3) the specificity of pesticides constructed on target insect α-amylases is lacking; and (4) research on insect α-amylases in artificial feed and in relation to human health is inadequate.

To facilitate the application of insect α-amylases, some measures need to be taken: Firstly, it is necessary to continue to strengthen the knowledge of different types of insect α-amylase and conduct basic research on α-amylase that can be helpful in deepening the understanding of the diversity, variability, and evolutionary relationship of α-amylase. Secondly, a database must be built for investigators to submit and summarize relevant knowledge of amylases and α-AIs, which is convenient for screening insect-specific α-AI, developing new non-target animal safety insecticide tools, and acquiring information regarding allergic reactions in humans or animals. Thirdly, artificial feeds must be developed and produced that can increase α-amylase activity and improve the production capacity of insects. Notably, the information gained from studies on the relationship between α-amylase activity and diapause can be utilized to develop artificial feeds that require diapause and prolong shelf life or to facilitate storage and transportation when used by natural enemies like parasitic wasps. This will strengthen the studies on the pathogenic mechanism of human allergy to insect α-amylase and the modification of insect food allergens. Finally, the efficacy and safety evaluations of plant amylase inhibitors in human obesity and diabetes also need to be improved.

Considering amylase’s multiple benefits for pest control, we hope this review will help to provide different perspectives for enriching our understanding of the potential and limitations of amylase in new pesticide development. In addition, these findings may facilitate the development of amylase as a potential alternative to current pest management strategies.

## Figures and Tables

**Table 1:** Location and function of non-gut amylase in insects.

Species	Location	Function	Reference
*Apis mellifera*	Hypopharyngeal gland	Converts nectar into honey	[35]
*Blattella germanica*	Tergal gland	Nuptial feeding stimulant	[11,38]
*Pteromalus puparum*	Muscle	Energy metabolism in muscle	[40,41]
*Pteromalus puparum*	Venom gland	Supports the development of offspring	[40,41]
*Cotesia flavipes*	Oral	Mediates host acceptance for oviposition	[42]

**Table 2:** Effects of different types of pesticides on amylase activity of target insects or non-target organisms.

Class	Sources	Target Insects	α-Amylase Activity	Non-Target Organism	α-Amylase Activity	References
Plant-derived extracts	Azukisapogenol triterpenoid saponins	*Acyrthosiphon pisum*	Decrease	-^1^	-	[99]
	*Magnolia grandiflora* (Magnoliaceae), *Schinus terebinthifolius* (Anacardiaceae), *Salix babylonica* (Salicaceae)	*Spodoptera littoralis*	Decrease	-	-	[100]
	Lectin extracted from *Polygonum persicaria* L. (PPA)	*Sitophilus oryzae*	Decrease	-	-	[101]
	Plumieride from *Himatanthus drasticus*	*Callosobruchus maculatus*	Decrease	-	-	[102]
	*Piper corcovadensis* leaf essential oil (PcLEO)	*Sitophilus zeamais*	Decrease	-	-	[103]
	*Artemisia annua* essential oil	*Glyphodes pyloalis*	Decrease	-	-	[104]
	Isoryanodane diterpenoid derived from *Itoa orientalis* (Ttol A)	*Spodoptera litura*	Decrease	-	-	[105]
	Sanguinarine in *Chelidonium majus*	*Lymantria dispar*	Decrease	-	-	[106]
	*Agave americana* leaf extract	*Sitophilus oryzae*	Decrease	-	-	[107]
	Toosendanin (TSN)	*Mythimna separata*	Invariability	-	-	[110]
Hormonal pesticide	Pyriproxyfen	*Musca domestica, citrus psyllids*, etc.	Decrease	*Bombyx mori*	Increase	[108]
	20-hydroxyecdysone	*Tribolium castaneum*	Decrease	*-*	-	[115]
Chemical pesticide	Phoxim	*Lepidoptera, Hemiptera*, etc.		*Bombyx mori*	Increase	[109]
	Spinetoram	*-*	-	*Bombus terrestris*	Decrease	[114]
	Glyphosate	*-*	-	*Bombus terrestris*	Decrease	[114]
	Neonicotinoids	*-*	-	*Apis mellifera*	Decrease	[116]
Antimicrobial peptides (AMPs)	γ-Thionin (BoT)	*Sitophilus oryzae* *Tribolium castaneum*	Decrease	-	-	[118]
RNAi	dsRNA	*Phenacoccus solenopsis*	Decrease	-	-	[111]
	dsRNA	*Anthonomus grandis*	Decrease	-	-	[112]
	dsRNA	*Helicoverpa armigera*	Decrease	-	-	[113]
Bactericide	*Bacillus thuringiensis*	*Callosobruchus maculatus*	Decrease	-	-	[117]

^1^ “-“in Table 2 means unreported.

## Data Availability

No new data were created or analyzed in this study. Data sharing is not applicable to this article.
